# Thyroid papillary carcinoma combined with primary follicular lymphoma: a case report

**DOI:** 10.1186/s13000-024-01495-0

**Published:** 2024-05-21

**Authors:** Ting Xu, Li Wu, Hua Ye, Shuai Luo, Jinjing Wang

**Affiliations:** 1 Department of Pathology, Affiliated Hospital of Zunyi Medical University, Zunyi City, Guizhou Province P.R. China; 2 Department of Pathology, Zhejiang Provincial People’s Hospital Bijie Hospital, Bijie City, Guizhou Province China

**Keywords:** EZH2 gene mutation, Hashimoto’s thyroiditis, Primary follicular lymphoma, Thyroid papillary carcinoma

## Abstract

**Background:**

Papillary thyroid carcinoma (PTC) stands out as the most prevalent epithelial malignant thyroid tumor. Thyroid primary follicular lymphoma (PFL) represents a rare malignant tumor originating from mesenchymal tissues. The concurrent occurrence of PTC and PFL is exceptionally rare, particularly in the context of Hashimoto’s thyroiditis, presenting significant challenges in clinical diagnosis and treatment.

**Case demonstration:**

A 44-year-old female patient presented with a neck mass persisting for over 1 month. The patient underwent surgery, and the incised tissues were subjected to pathology examinations, along with immunohistochemistry and next-generation sequencing tests suggestive of an EZH2 gene mutation in the tumor cells. The final pathological diagnosis confirmed the presence of PTC combined with PFL. Following a 27-month follow-up, the patient displayed no signs of recurrence or metastasis.

**Conclusions:**

The concurrent occurrence of PTC and PFL poses notable challenges in clinical practice, requiring careful consideration in diagnosis and treatment. Herein, we present a rare case of PTC combined with PFL featuring an EZH2 gene mutation, which can be easily overlooked in the context of Hashimoto’s thyroiditis. The patient’s favorable response to surgical and radiotherapeutic interventions underscores the importance of accurate diagnosis and tailored treatment strategies in similar cases.

**Supplementary Information:**

The online version contains supplementary material available at 10.1186/s13000-024-01495-0.

## Background

Papillary thyroid carcinoma (PTC) is a prevalent malignant tumor of the thyroid, whereas thyroid primary follicular lymphoma (PFL) is a rare tumor typically associated with chronic lymphocytic thyroiditis (Hashimoto’s thyroiditis). The concurrent occurrence of two tumors of different origins in the same location is exceedingly rare [1] and presents a diagnostic challenge. Both tumors often develop in the context of Hashimoto’s thyroiditis, further complicating diagnosis [2]. Although there are case reports of thyroid diffuse large B-cell lymphoma combined with PTC [3] and thyroid mucosa-associated lymphoid tissue lymphoma combined with PTC [4], documented cases of PFL combined with PTC are scarce. Follicular lymphoma, categorized as a small B-cell lymphoma, can be challenging to distinguish from Hashimoto’s thyroiditis in pathological diagnosis. Herein, we present a unique case of PTC combined with PFL.

## Case demonstration

**Fig. 1:**
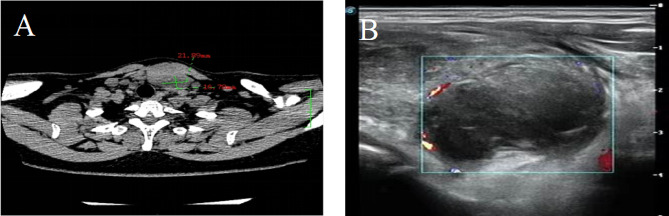
**A** - Computerized tomography (CT) scan showing a low-density mass measuring 2.1 cm × 1.7 cm in the left thyroid lobe. **B** - Thyroid ultrasound revealing a solid hypoechoic nodule measuring approximately 2.0 cm × 1.8 cm with unclear borders, irregular shape, and non-uniform internal echogenicity in the left thyroid lobe

A 44-year-old female patient presented with a neck mass persisting for over 1 month with occasional difficulty breathing. Upon examination, a palpable circular mass with a tough texture and clear boundaries measuring 3 cm × 2 cm in size was found near the isthmus of the left thyroid lobe. The mass was immovable but showed upward and downward movement during swallowing. No obvious nodules were palpable in the right thyroid lobe or isthmus; auscultation revealed no vascular murmur. The patient did not experience fever, weight loss, or night sweats and had no history of thyroid disease, immunodeficiency, or malignancy. Palpation showed no enlarged superficial lymph nodes. Serological examination indicated TG-Ab of 7.7 IU/L and TPO-Ab of > 950.00 IU/L. Thyroid B-scan ultrasonography revealed an increase in the thyroid volume, decreased parenchymal echogenicity, a low-echogenicity nodule in the left lobe classified as TI-RADS category 3 (Fig. 1A); computerized tomography (CT) scan showed a low-density mass measuring 2 cm × 1.8 cm in the left thyroid (Fig. 1B). The large mass had compressed the bronchus and caused dyspnea, and the symptoms of dyspnea did not improve significantly after oxygen inhalation. Therefore, surgery was performed 4 days after admission. During the surgery, three masses of different sizes were observed on the left thyroid gland. The larger one was approximately 3 cm × 2 cm in size, located at the lower pole of the left lobe of the thyroid gland, with tough texture and unclear boundary. A malignant tumor of the left lobe of the thyroid was diagnosed. Total resection of the remaining thyroid, lymph node dissection in zone IV, and parathyroid transplantation were performed.

Pathological gross examination of the left thyroid revealed a piece of thyroid tissue measuring 7.5 cm × 5.0 cm × 3.5 cm, with a smooth surface, intact capsule, and had focus, along with a 2-cm diameter gold, solid, hard nodule evident on the cut surface.

**Fig. 2:**
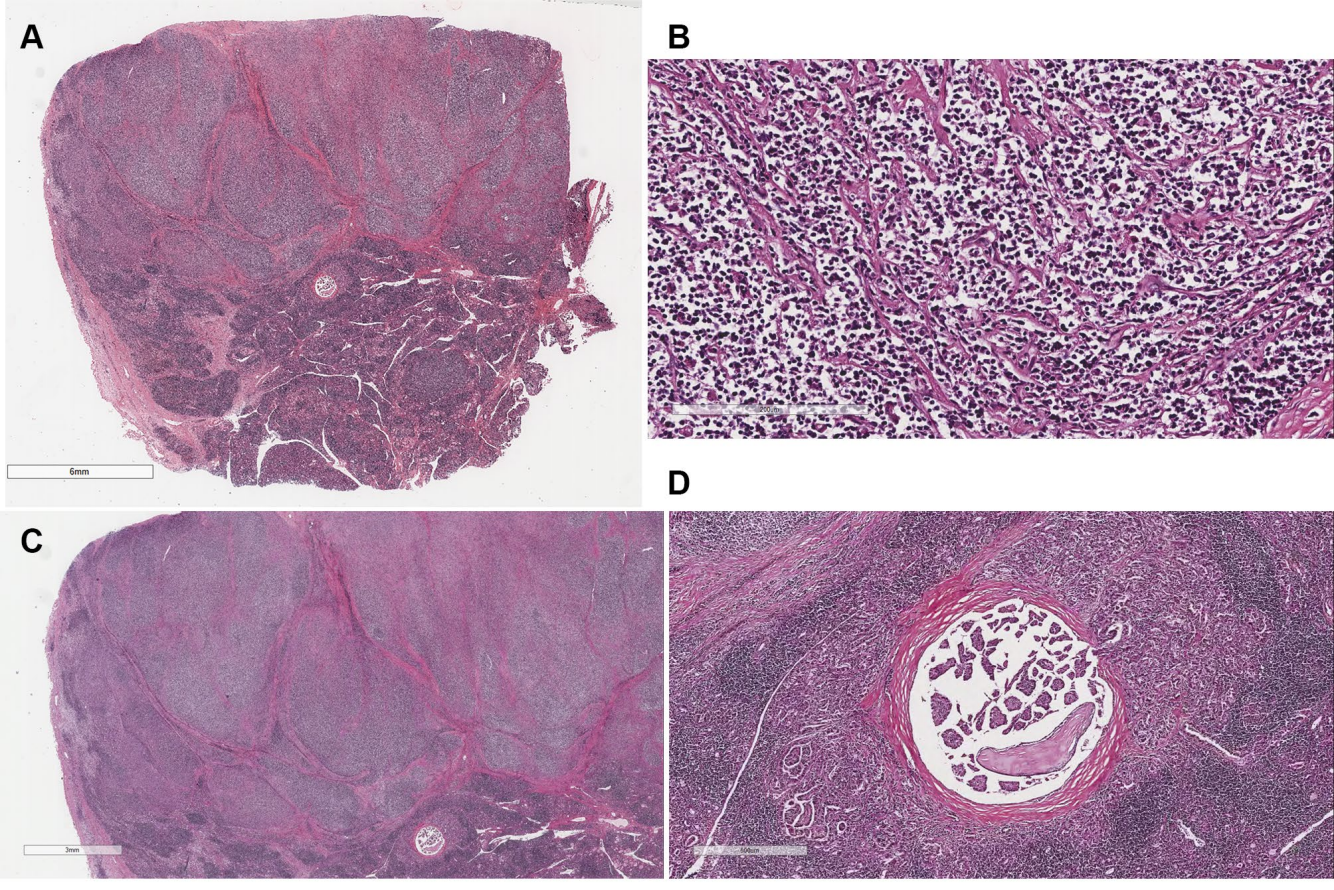
**A** - Follicular lymphoma cells stained with hematoxylin–eosin (HE), 50× magnification. **B** - Follicular lymphoma cells stained with HE, 200× magnification. **C** - In the context of Hashimoto’s thyroiditis, abundant lymphocyte infiltration and lymphoid follicle formation were observed in the interstitium, along with atrophy of thyroid follicular epithelium and eosinophilic degeneration.HE, 10× magnification. **D**  D-D-PTC cells stained with HE, 20× magnification

**Fig. 3:**
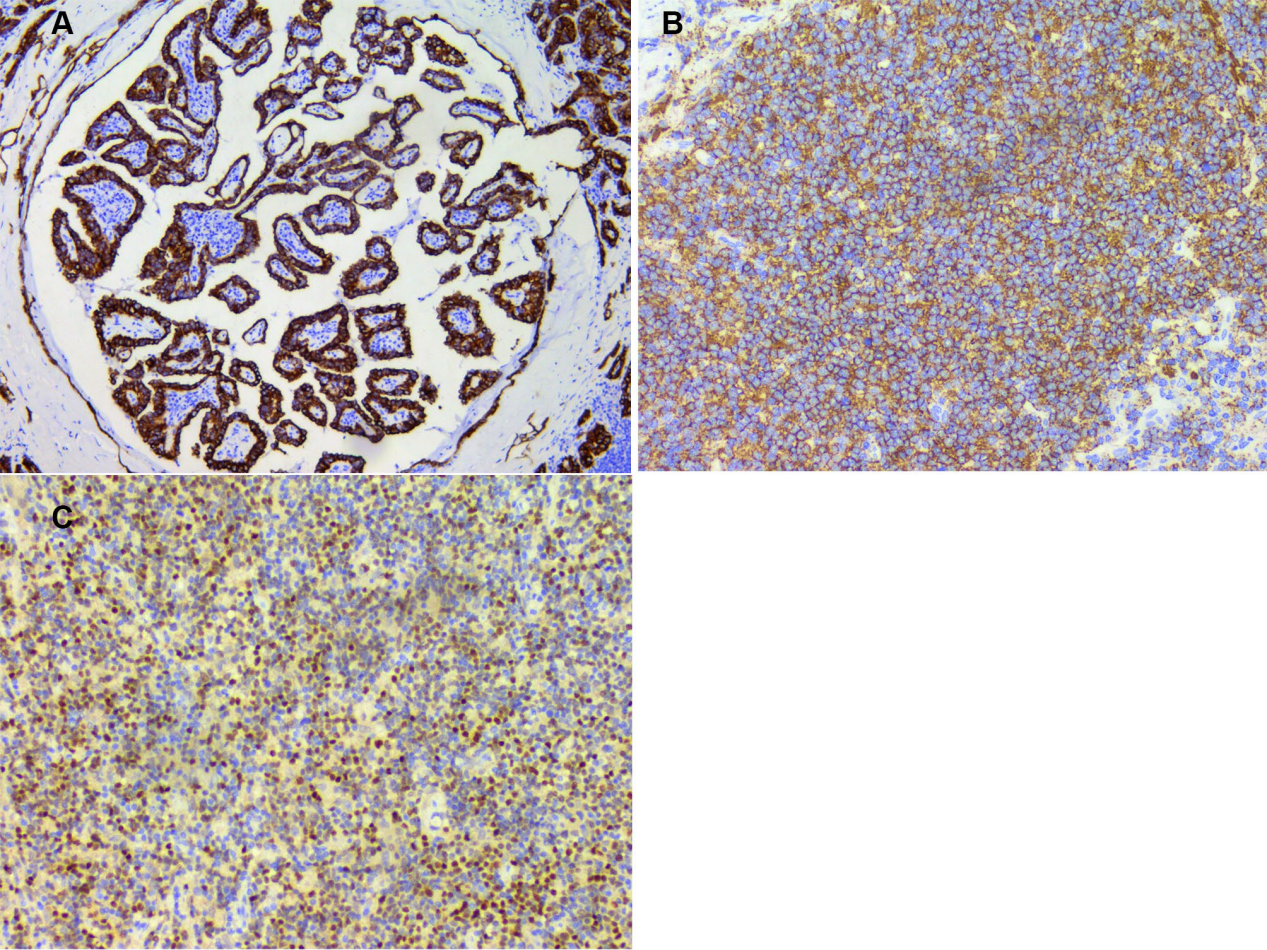
**A** - Pan-cytokeratin (CK19) positivity under immunohistochemical staining, 200× magnification. **B** CD20 positivity under immunohistochemical staining, 200× magnification. **C** - Bcl-6 positivity under immunohistochemical staining, 200× magnification

Histopathological examination revealed follicles of relatively uniform size arranged tightly with sclerotic bands under low magnification (Fig. 2A). Under high magnification, the tumor cells consisted of diffuse, small, round, polygonal tumor cells with little cytoplasm. Most nuclei displayed irregular shapes, with some being round, characterized by clear nuclear membranes and slightly coarse chromatin (Fig. 2B). In the context of Hashimoto’s thyroiditis, abundant lymphocyte infiltration and lymphoid follicle formation were observed in the interstitium, along with atrophy of thyroid follicular epithelium and eosinophilic degeneration (Fig. 2C). Additionally, focal areas exhibited the histopathological features of PTC (tumor diameter of 2.26 mm), including irregular nuclear membranes, large and crowded nuclei resembling ground glass, and intranuclear inclusions (Figs. 2D and 3).

Immunohistochemistry findings indicated positivity for several markers, including pan-cytokeratin, CK19, galectin3, HBME3, TG, TTF1, PAX8, CD56, and Ki67 (positivity rate of 3%), and negativity for CT, in the tumor cells in PTC. Immunohistochemical findings of tumor cells in PFL were as follows: LCA+, CD20+, CD79a+, bcl6+, CD10 (+), PAX5+, bcl2+, CD23/CD21 (follicular dendritic cell mesh was present and disrupted), MUM-1-, CD3-, CD5-, p53(wild type). 60% positivity rate for Ki67.

Next-generation sequencing, where 730 gene mutations such as KRAS, BRAF, and PIK3CA were detected by second-generation sequencing method based on target region capture, revealed an EZH2 gene mutation in the tumor cells. Regarding gene function, the EZH2 gene encodes a histone methyltransferase. As a member of the PcG family, EZH2 has the effect of inhibiting gene transcription and is involved in embryonic development and cell differentiation. EZH2 gene mutations are found in myelodysplastic syndrome (MDS), lymphoma, colorectal cancer, and endometrial cancer. EZH2 can inhibit the transcription of tumor suppressor genes, and EZH2 overexpression or activation mutation is found in a variety of malignant tumors except melanoma, such as breast cancer, prostate cancer, uterine cancer, gastric cancer, and nuclear renal cell carcinoma. The tyrosine mutation at code 646 of the EZH2 gene is phenylalanine. Y646 is a common variant site of EZH2 in Hodgkin’s lymphoma. In vitro experiments showed that Y646F mutation can change the substrate specificity of the EZH2 protein, improve the catalytic efficiency of H3K27 trimethylation, and promote tumor progression. According to the available evidence, EZH2:c.1937 A > T; p.Y646F is considered tumorigenic 3.

The final pathological diagnosis showed PTC of the classic type according to the 5th edition of WHO Classification criteria for thyroid tumors in 2022 combined with PFL involving the left thyroid and isthmus.

Postoperative positron emission tomography-CT whole-body scans showed no evidence of neck involvement or organ metastasis. Post-surgery, regular oral levothyroxine sodium tablets have been administered since (75 µg, oral Qd). After completing relevant examinations, the patient was diagnosed with grade 3 A, stage IIEA thyroid follicular lymphoma. After exclusion of the conjunctivitis conjunctivitis, radiotherapy was performed in the thyroid operative area and bilateral middle and lower neck lymph node drainage area after discharge, with DT = 36 Gy/20f and a course of 12 months. After radiotherapy, oral mucositis of grade II appeared, which was improved after anti-inflammatory and other symptomatic treatments. Imaging reexamination showed that the curative effect was evaluated as CR.

Serological examination after 1 month of radiotherapy (5 thyroid function items: T3: 1.21 nmol/L; T4: 64.90 nmol/L; FT3: 3.06 pmol/L; FT4: 10.30 pmol/L; and TSH: 7.93mIU/L) indicated the presence of complications of hypothyroidism. After a 27-month follow-up, the patient remained free from recurrence or metastasis.

## Discussion

PTC stands as the most prevalent malignant tumor of follicular cell origin among thyroid tumors, representing 80-85% of all cases. It exhibits a predilection for females and can manifest at any age. Its occurrence in patients with Hashimoto’s thyroiditis is prevalent [5]. However, the exact pathogenesis remains elusive; prolonged Hashimoto’s thyroiditis may elevate TSH levels, potentially acting as a growth factor for malignant tumors [6]. In patients with Hashimoto’s thyroiditis, PTC is typically detected at a younger age, characterized by smaller nodules and later TNM staging, often devoid of local or systemic invasion [7–9]. The coexistence of PTC and Hashimoto’s thyroiditis often results in smaller nodules that are easily overlooked.

PTL presents as a rare heterogeneous tumor [10]. Lymphomas arising in the thyroid can be classified as primary or secondary. PTL refers to malignant tumors originating from the thyroid’s lymphoid tissue, constituting approximately 2–5% of extranodal non-Hodgkin lymphomas [11]. The most common subtype is diffuse large B-cell lymphoma, accounting for approximately 50% of all cases, followed by mucosa-associated lymphoid tissue lymphoma, accounting for approximately 10–23% of all cases [12]. Thyroid PFL accounts for approximately 1–6% of all PTL cases [13].

The clinical manifestations of PFL combined with PTC vary, typically featuring a rapidly enlarging, painless thyroid mass. Smaller masses may be asymptomatic, while larger masses can lead to compressive symptoms such as dyspnea, wheezing, dysphagia, and hoarseness. Diagnostic imaging modalities like thyroid ultrasound and CT scans offer valuable insights, but definitive diagnosis necessitates postoperative pathological examination. The pathogenesis of PTL remains unclear, with some associations suggested with viral infections and immune deficiencies. Hashimoto’s thyroiditis, an autoimmune disorder [14], is considered a risk factor and underlying cause of PTL. Patients are predominantly female, with a wide age range [15]. The incidence of PTL is significantly increased by 67–80 times in individuals with Hashimoto’s thyroiditis, with a male-to-female ratio as high as 1:20 [16].

Advancements in next-generation sequencing have shed light on frequent mutations in epigenetic regulators in follicular lymphoma, with the EZH2 gene being a notable target [17]. Approximately 25% of follicular lymphomas harbor EZH2 mutations, which are associated with a favorable prognosis [18]. Patients with EZH2 mutations have garnered attention in the realm of epigenetic therapy.

Distinguishing PTC from Hashimoto’s thyroiditis poses challenges. In Hashimoto’s thyroiditis, residual follicular epithelium may display atypia, characterized by enlarged and crowded cell nuclei, irregular nuclear contours, and the presence of nuclear grooves, complicating its differentiation from PTC. Immunomarkers such as Hector Battifora mesothelial-1 and cytokeratin 19 can aid in differentiation, as they are expressed in PTC but not in Hashimoto’s thyroiditis, facilitating accurate diagnosis.

Regarding distinguishing Hashimoto’s thyroiditis from PFL, the thyroid capsule thickens, and the proliferating lymphocytes do not disrupt the capsule and replace the normal thyroid tissue in Hashimoto’s thyroiditis. The proliferating lymphocytes are mature lymphocytes, and the remnant thyroid follicular epithelium may become eosinophilic. By contrast, PFL presents with relatively uniform follicles, some of which may fuse together. The proliferating lymphocytes in PFL exhibit monoclonal proliferation. Additionally, tumor cells are heterogeneous, which disrupt the thyroid capsule and infiltrate into the peri-thyroid tissue [19]. Immunophenotypically, PFL resembles nodal germinal centers, expressing CD20+, CD23+, CD10+, BCL-2+/-, and Bcl-6+.

Numerous clinical and pathological studies indicate that PTC [5, 19] and lymphoma [1, 3, 6, 7, 11, 19] can occur in the context of Hashimoto’s thyroiditis. However, the concurrent occurrence of these two malignant tumors in the context of Hashimoto’s thyroiditis is rare [20]. PTC generally carries a favorable prognosis and can be effectively managed with surgical resection. By contrast, postoperative treatment of lymphoma may involve local radiotherapy, multidrug chemotherapy, immunotherapy, or a combination of radiotherapy and chemotherapy, depending on histological subtypes and staging. Pathologists should avoid settling for a single diagnosis to prevent misdiagnosis and ensure accurate diagnosis for precise clinical treatment.

## Conclusion

The concurrent occurrence of PTC and lymphoma is relatively uncommon, presenting certain challenges in clinical diagnosis and treatment. We reported a case of PTC combined with PFL featuring an EZH2 gene mutation. The patient underwent surgical treatment and radiotherapy, with the current treatment showing positive outcomes. This case provides valuable insights for clinical physicians in the diagnosis and treatment of similar cases.

### Electronic supplementary material

Below is the link to the electronic supplementary material.

Supplementary Material 1

## Data Availability

No datasets were generated or analysed during the current study.

## Abbreviations

CT: Computed tomography
PTC: Papillary thyroid carcinoma
PFL: Primary follicular lymphoma
