# Supplementary material for: Thyroid papillary carcinoma combined with primary follicular lymphoma: a case report
Source: Diagn Pathol. 2024 May 21;19:69. doi: 10.1186/s13000-024-01495-0 (PMC11107012; doi:10.1186/s13000-024-01495-0)
Supplement: Supplementary file 1 — Supplementary Material 1 [file 13000_2024_1495_MOESM1_ESM.pdf]

CARE
case report guidelines

CARE Checklist of information to include when writing a case report

(cc) BY-NC-ND

Topic	Item	Checklist item description	Reported on Line
Title	1	The diagnosis or intervention of primary focus followed by the words "case report"	Line 1 to 2 on page 1
Key Words	2	2 to 5 key words that identify diagnoses or interventions in this case report, including "case report"	Line 21 on page 1
Abstract (no references)	3a	Introduction: What is unique about this case and what does it add to the scientific literature?	Line 7 to 20 on page 1
	3b	Main symptoms and/or important clinical findings	Line 7 to 20 on page 1
	3c	The main diagnoses, therapeutic interventions, and outcomes	Line 7 to 20 on page 1
	3d	Conclusion—What is the main "take-away" lesson(s) from this case?	Line 7 to 20 on page 1
Introduction	4	One or two paragraphs summarizing why this case is unique (**may include references**)	Line 22 to 27 on page 2
Patient Information	5a	De-identified patient specific information	N/A
	5b	Primary concerns and symptoms of the patient	Line 4 to 9 on page 2
	5c	Medical, family, and psycho-social history including relevant genetic information	Line 4 to 9 on page 2
	5d	Relevant past interventions with outcomes	N/A
Clinical Findings	6	Describe significant physical examination (PE) and important clinical findings	Line 6 to 10 on page 2
Timeline	7	Historical and current information from this episode of care organized as a timeline	Line 4 to 10 on page 2
Diagnostic Assessment	8a	Diagnostic testing (such as PE, laboratory testing, imaging, surveys).	Line 4 on 10 page 2
	8b	Diagnostic challenges (such as access to testing, financial, or cultural)	Line 1 on page 3
	8c	Diagnosis (including other diagnoses considered)	Line 1 on page 3
	8d	Prognosis (such as staging in oncology) where applicable	Line 9 on page 3
Therapeutic Intervention	9a	Types of therapeutic intervention (such as pharmacologic, surgical, preventive, self-care)	Line 3 to 8 on page 3
	9b	Administration of therapeutic intervention (such as dosage, strength, duration)	Line 3 to 8 on page 3
	9c	Changes in therapeutic intervention (with rationale)	Line 3 to 8 on page 3
Follow-up and Outcomes	10a	Clinician and patient-assessed outcomes (if available)	N/A
	10b	Important follow-up diagnostic and other test results	N/A
	10c	Intervention adherence and tolerability (How was this assessed?)	N/A
	10d	Adverse and unanticipated events	N/A
Discussion	11a	A scientific discussion of the strengths AND limitations associated with this case report	Line 10 to 28 on page 3
	11b	Discussion of the relevant medical literature **with references**	Line 1 to 28 on page 12
	11c	The scientific rationale for any conclusions (including assessment of possible causes)	Line 12 to 23 on page 10
	11d	The primary "take-away" lessons of this case report (without references) in a one paragraph conclusion	Line 12 to 23 on page 10
Patient Perspective	12	The patient should share their perspective in one to two paragraphs on the treatment(s) they received	N/A
Informed Consent	13	Did the patient give informed consent? Please provide if requested	Yes ☑ No ☐
